# Prenatal exposure to polybrominated diphenyl ethers and BMI Z-scores from 5 to 14 years

**DOI:** 10.1186/s12940-022-00893-5

**Published:** 2022-09-08

**Authors:** Allison Kupsco, Andreas Sjödin, Whitney Cowell, Richard Jones, Sharon Oberfield, Shuang Wang, Lori A. Hoepner, Dympna Gallagher, Andrea A. Baccarelli, Jeff Goldsmith, Andrew G. Rundle, Julie B. Herbstman

**Affiliations:** 1 Department of Environmental Health Sciences, Mailman School of Public Health, Columbia University, 722 W 168th St. Room 1105, New York, NY 10032 USA; 2 Division of Laboratory Sciences, National Center for Environmental Health, Centers for Disease Control and Prevention, Atlanta, GA USA; 3 Department of Environmental Medicine and Public Health, Icahn School of Medicine at Mount Sinai, New York, NY USA; 4 Division of Pediatric Endocrinology, Diabetes and Metabolism, Department of Pediatrics, New York-Presbyterian Morgan Stanley Children’s Hospital, New York, NY USA; 5 Department of Biostatistics, Mailman School of Public Health, Columbia University, New York, NY USA; 6 Department of Environmental and Occupational Health Sciences, School of Public Health, SUNY Downstate Health Sciences University, Brooklyn, NY USA; 7 Nutrition Obesity Research Center, Columbia University Irving Medical Center, New York, NY USA; 8 Department of Epidemiology, Mailman School of Public Health, Columbia University, New York, NY USA

**Keywords:** Polybrominated diphenyl ethers, PBDEs, children’s environmental health, Adiposity, BMI, Prenatal exposures

## Abstract

**Background:**

Polybrominated diphenyl ethers (PBDEs) are flame-retardant compounds widely used in household products until phase out in 2004. PBDEs are endocrine disruptors and are suggested to influence signaling related to weight control. Prenatal exposures to PBDEs may alter childhood adiposity, yet few studies have examined these associations in human populations.

**Methods:**

Data were collected from a birth cohort of Dominican and African American mother-child pairs from New York City recruited from 1998 to 2006. PBDE congeners BDE-47, − 99, − 100, and − 153 were measured in cord plasma (ng/μL) and dichotomized into low (< 80th percentile) and high (>80th percentile) exposure categories. Height and weight were collected at ages 5, 7, 9, 11, and an ancillary visit from 8 to 14 years (*n* = 289). Mixed-effects models with random intercepts for participant were used to assess associations between concentrations of individual PBDE congeners or the PBDE sum and child BMI z-scores (BMIz). To assess associations between PBDEs and the change in BMIz over time, models including interactions between PBDE categories and child age and (child age)^2^ were fit. Quantile g-computation was used to investigate associations between BMIz and the total PBDE mixture. Models were adjusted for baseline maternal covariates: ethnicity, age, education, parity, partnership status, and receipt of public assistance, and child covariates: child sex and cord cholesterol and triglycerides.

**Results:**

The prevalence of children with obesity at age 5 was 24.2% and increased to 30% at age 11. Neither cord levels of individual PBDEs nor the total PBDE mixture were associated with overall BMIz in childhood. The changes in BMIz across childhood were not different between children with low or high PBDEs. Results were similar when adjusting for postnatal PBDE exposures.

**Conclusions:**

Prenatal PBDE exposures were not associated with child growth trajectories in a cohort of Dominican and African American children.

**Supplementary Information:**

The online version contains supplementary material available at 10.1186/s12940-022-00893-5.

## Introduction

Childhood obesity is a major public health issue that results in a hefty burden of adult disease [1, 2]. This increase cannot be explained by lifestyle and genetic factors alone and environmental toxicants are increasingly recognized as contributors to obesity [3, 4]. Prenatal development is a precise spaciotemporal process that is particularly susceptible to environmental insult. Prenatal environmental exposures can cause long-lasting structural and functional changes in cells, organs, and tissues [4–6] that may lead to altered adiposity phenotypes in childhood. Polybrominated diphenyl ethers (PBDEs) are endocrine disrupting compounds that were produced as a mixture to satisfy fire-safety regulations. Around 85% of PBDEs were produced for use in the US, resulting in the highest exposures worldwide [7]. Although PBDEs were voluntarily phased out of production in 2004, exposure continues due to infrequent replacement of PBDE-containing products and a long environmental half-life. It is anticipated that PBDEs present in consumer products disposed of in landfills will migrate into the food chain, resulting in new exposure sources and greater future public health concerns [8, 9]. As endocrine disrupting compounds, PBDEs can interfere with normal signaling related to weight control [3, 10, 11]. Several murine studies of perinatal PBDE exposure demonstrate that PBDEs increase body weight [12–15], and alter adipokines, carbohydrates, lipids, and steroid metabolism [16]. In vitro, PBDEs induce adipocyte differentiation and increase adipokine gene expression [17, 18].

To date, few human studies have examined associations of adiposity with prenatal PBDE levels with mixed results. Examination of prenatal PBDEs in 318 children found BDE-153 and total PBDEs negatively associated with BMI and waist circumference from ages 2 to 8 [19]. However, a second study examining environmental mixtures on child BMI at age 7 found non-significant negative associations with PBDEs [20]. Hence, experimental and epidemiological research suggest opposing roles for prenatal PBDEs in adiposity; yet further analysis in human cohorts is needed to elucidate these effects. Furthermore, low-income minority communities who may be less likely to replace older PBDE containing furniture and more likely to purchase used furniture, have excess risk for continued exposure, highlighting a critical role for PBDEs in health disparities [21, 22]. The present analysis focuses on a cohort of African American and Dominican mother-child pairs from New York City; children of these ethnicities have particularly high obesity prevalence, increasing from 5% in 1980 to 22% in 2014 [1, 2]. Our objective was to determine the association between congener-specific prenatal PBDE exposures or the total PBDE mixture and childhood adiposity and growth across childhood and examine sex-specific and race/ethnicity-specific associations. We hypothesized that prenatal PBDE exposures would be associated with a steeper increase and higher overall levels of BMIz throughout childhood.

## Materials and methods

### Study sample

This analysis used participant information and samples from the Columbia Children’s Center for Environmental Health (CCCEH) Mothers and Newborns Cohort, which recruited women from Northern Manhattan and the South Bronx, New York, between 1998 and 2006. Enrollment details are described elsewhere [23]. In brief, 727 women were enrolled in the third trimester of pregnancy at New York-Presbyterian Hospital and Harlem Hospital Center. Women between the ages of 18 and 35, who self-identified as African American or Dominican, and resided in Northern Manhattan/Bronx for at least one year were considered eligible for the study. Women were excluded if they were current smokers at recruitment, reported illicit drug use, or had a diagnosis of HIV, diabetes, or hypertension. We excluded a further four participants with infant cord cotinine levels > 25 ng/mL from this analysis, which likely indicated active smoking near the time of birth [24, 25]. All study protocols were approved by the Institutional Review Board of Columbia University and the Centers for Disease Control and Prevention. Mothers were informed about all study procedures before each visit and provided written informed consent to participate. Children provided informed assent beginning at age 7.

### Data collection

During a prenatal visit in the third trimester of pregnancy and at each follow-up visit, trained bilingual research workers conducted interviews to determine information on maternal demographics, smoking history, income, education level, receipt of public assistance, height, and pre-pregnancy weight. Infants’ sex and birthweight was obtained from medical records after delivery. Children returned for follow-up visits at ages 5, 7, 9, and 11 years during which trained research workers collected height and weight data. An ancillary study also collected data from subsets of the cohort children between ages 9.2 and 14.3 (TAPAS I) years and again between ages 11.3 and 14.5 years (TAPAS III). Anthropometric data were collected using the same standardized protocol as previously described [26]. At age 5, weight was measured to the nearest 0.1 kg using a Detecto Cardinal 750 digital scale while the child was wearing light clothes and no shoes and from age 7 onwards weight was measured using a Tanita scale (Model BC-418). The Centers for Disease Control and Prevention’s SAS macro was used to calculate BMI Z-score (BMIz) and BMI percentiles at each follow-up visit based on the CDC 2000 Growth Chart reference sample. Not all children attended each follow-up visit; 259 children attended all five potential visits, 126 attended four visits, 85 attended three visits, 45 attended two visits, and 26 attended one visit. Full details on the visits attended and skipped for the entire study population and the analysis subset can be found in Supplemental Table S1.

### Measurement of cord serum PBDEs

Umbilical cord blood and peripheral blood at age 7 were collected by trained research staff (at delivery) or a trained pediatric phlebotomist (during childhood). Blood was processed and stored at − 80 °C at the CCCEH repository for later analysis. Sample aliquots of 1 mL of plasma were sent to the CDC for analysis of 11 PBDE congeners (BDEs: 17, 28, 47, 66, 85, 99, 100, 153, 154, 183, and 209). The present study examines BDEs − 47, − 99, − 100, and − 153, which were the most frequently detected congeners across study visits and are the most prevalent congeners representing 90% of the human body burden [27]. The analytical method has been previously described [28]. In brief, samples were processed using automatic fortification with internal standards and extracted with automated liquid-liquid extraction (Gilson Inc.; Middleton, WI). Gas chromatography isotope dilution high resolution mass spectrometry (GC-MS) was used for analytical determinations. We determined total cholesterol and triglyceride levels using commercially available standardized enzymatic kits (Roche Diagnostics; Indianapolis, IN). Wet weight PBDE measures were not standardized a priori for lipid levels, as we reasoned that cord lipids may act as a confounder between PBDEs and childhood adiposity. Instead, total cholesterol and triglycerides were included as covariates in all statistical models. Infants with a measure of PBDEs available for analysis weighed more at birth and were more often born to a nulliparous mother [29].

### Statistical analysis

As a large number of samples had values below the limits of detection (BDE-47: 20%, BDE-99: 50%, BDE-100: 60% and BDE-153: 64%), we used a distribution-based method for multiple imputation that incorporated sample-specific limits of detection to impute 10 datasets for natural-log transformed PBDEs as previously described [29]. We selected this approach over imputation with a constant, such as the LOD/2 or zero, because it provides less bias over a wide range of the percent missingness and also meets linear modeling assumptions [30]. We next calculated a sum PBDE measure as the molar sum of the four compounds. PBDEs were subsequently modeled continuously or categorized into dichotomous variables with children having concentrations greater than the 80th percentile categorized as “high” and those at or below the 80th percentile categorized as “low” to ensure that all participants within the “high” category had values above the detection limits and that our categories were large enough for stratified analyses. Child BMI z-scores (BMIz) were modeled continuously, and we used CDC percentiles to categorize children as experiencing either “under or normal weight” (<85th percentile), “overweight” (85th to 95th percentile), or “obesity” (>95th percentile). As only three children could be categorized as underweight (<5th percentile), we combined normal and underweight into a single category. Overall, 289 children had PBDE measurements available in cord blood and returned for at least one follow-up visit.

We selected covariates hypothesized to predict both child BMI and prenatal PBDE exposure based on previous studies [26, 29], and included maternal characteristics collected at birth: maternal parity (primiparous/multiparous), age at birth, ethnicity (Dominican/African American), receipt of public assistance (yes/no), completed high school (yes/no), partnership status (partnered/single), as well as the childhood variables of linear and quadratic child age at visit (centered at age 5) and child sex in models. Of these, 7 mothers were missing pre-pregnancy BMI, which we imputed using multiple imputations of chained equations (mice) using predictive mean matching in the mice package in R [31]. To maximize the amount of available information, data from all nonsmoking 702 participants were utilized for imputation [32]. We generated 10 multiply imputed pre-pregnancy BMI variables and used maternal variables during pregnancy and child birthweight, gestational age, and BMI at age 5 to impute the missing data. Maternal variables collected at birth were ethnicity, maternal age, parity, born in the US, environmental tobacco smoke at birth, pre-pregnancy weight, maternal height, receipt of public assistance, report of material hardship, education. Continuous covariates were centered and scaled for modeling.

To examine associations between prenatal PBDE concentrations and overall childhood BMIz, we ran mixed-effects models for BMIz with a random intercept for subject. Separate models were specified for each PBDE as PBDEs are highly correlated. Two sets of models were run, adjusting for 1) minimal covariates (sex and child age and age^2^) or 2) all covariates. Next, we investigated associations between cord PBDE levels and child BMIz over time by including interactions between each categorical PBDE and child age and age^2^ at each BMI measurement in our mixed-effects models. Interpreting the effect estimates and intercepts of these models allow us to determine the growth curves for individuals in each PBDE category. As previous research has suggested that the relationship between child BMIz and age is quadratic [26], to determine whether potential interactions between age and PBDEs on BMIz were nonlinear, we fit generalized additive mixed-models with child age and a random intercept for participant (data not shown). As these models indicated a nonlinear relationship, for simplicity of interpretation, we fit a series of nested models including age* PBDE category, age^2^* PBDE category, or age^3^* PBDE category. Tests comparing these models found that the age^2^*PBDE category was a significantly better fit than models containing age*PBDE category or age^3^*PBDE category (*p* < 0.05). Associations were analyzed with age at BMIz measurement offset by − 60 months (5 years) so that regression coefficients for high prenatal exposure could be interpreted as the difference in BMIz at age 5 years compared to individuals with low prenatal exposure. Trajectories could not be calculated for children that attended only one visit. As previous studies have suggested that BMI may be more reliable in studies evaluating adiposity overtime, we opted to include an additional analysis of trajectories specifying BMI as the outcome instead of BMIz [33–35]. Finally, we repeated models stratified by child sex to determine sex-specific associations and stratified by race/ethnicity to determine race/ethnicity-specific associations.

We used quantile g-computation to examine the associations between the total PBDE mixture and longitudinal BMIz [36]. Quantile g-computation calculates a weighted sum of all exposures based on quantiles, then estimates the effect of increasing all exposures by one quantile simultaneously on the outcome, conditional on covariates, using a generalized linear mixed model implementation of g-computation with a random intercept for subject. We examined BDE-47, BDE-99, BDE-100, and BDE-153 simultaneously as quartiles and ran 1000 bootstraps to estimate confidence bounds of the total mixture effect (Ψ). This can be interpreted as the effect of increasing the total PBDEs by one quartile on child BMIz. We then estimated the weight of each PBDE to the overall association.

Results from analyses with multiply imputed data were combined using Rubin’s rules [37]. All statistical analyses were performed in R version 4.1 [38].

### Sensitivity analyses

To determine the robustness of our findings, we performed several sensitivity analyses. First, to account for possible bias due to differential loss-to-follow-up, we calculated inverse probability weights for each follow-up visit using the *ipw* package in R [39]. Weights were generated using logistic regression models for successful follow-up at each visit predicted by variables that were significantly associated with follow-up: PBDEs, cord triglycerides and cholesterol, maternal age, maternal BMI, maternal parity, and maternal ethnicity. Separate inverse probability weights were generated for each PBDE and included in PBDE-specific mixed-effects models. To determine the role of lipid-adjustment on our findings, we also performed sensitivity analyses using PBDEs standardized for total lipids as previously described [29]. Next, we further adjusted models for PBDEs measured at the year 7 visit in a subset of the population (*n* = 194) to explore the likelihood that postnatal confounding could be driving our results. These models were fit with and without the inclusion of the year-5 visit, which did not alter the findings. Finally, to ensure that our results were robust to our chosen dichotomization of “high” and “low” PBDE values at the 80th percentile, we re-ran our adjusted models (Model 2) including PBDEs dichotomized at lower and higher thresholds: the 65th percentile (to ensure that all were above the 65% < LOD for PBDE-153) and the 90th percentile.

## Results

Of the children with cord serum PBDEs measured, 289 attended at least one follow-up visit during the study period: 260 attended the 5-year visit, 266 attended the 7-year visit, 242 attended the 9-year visit, 210 attended the 11-year visit, and 209 attended the ancillary TAPAS visits. Of those 153 attended all five visits, 124 attended between two and four visits, and 12 attended only one visit, with most children with fewer visits attending only the earlier visits (Supplemental Table S1). The intraclass correlation coefficient for BMIz was 0.84. The prevalence of obesity was 24.3% at age 5 and increased to 30.0% at age 11 (Table 1).

**Table 1:** Characteristics of children at each follow-up visit included in the analysis and maternal characteristics of children that followed up at each visit as mean (standard deviation) or N (percent)

Child characteristics at each visit	5 Year Visit (*n* = 260)	7 Year Visit (*n* = 266)	9 Year Visit (*n* = 242)	11 Year Visit (*n* = 210)	8–14 Year Visit^a^ (*n* = 209)
Age in Months	60.6 (2.3)	84.8 (2.19)	109 (2.78)	133 (4.49)	121 (13.44)
BMI Z-score	0.71 (1.34)	0.90 (1.15)	0.99 (1.1)	0.96 (1.12)	1.1 (1.08)
Obesity Status					
Not with Obesity	197 (75.8)	198 (74.4)	166 (68.6)	147 (70.0)	129 (61.7)
With Obesity	63 (24.2)	68 (25.6)	76 (31.4)	63 (30.0)	80 (38.3)
Race/Ethnicity					
African American	101 (38.8)	101 (38)	95 (39.3)	79 (37.6)	85 (40.7)
Dominican	159 (61.2)	165 (62)	147 (60.7)	131 (62.4)	124 (59.3)
Sex					
Female	145 (55.8)	145 (54.5)	132 (54.5)	116 (55.2)	112 (53.6)
Male	115 (44.2)	121 (45.5)	110 (45.5)	94 (44.8)	97 (46.4)
Maternal characteristics at birth for the sub-sample of children within each visit period					
Maternal Age (years)	25.3 (4.91)	25.4 (4.86)	25.4 (4.94)	25.5 (5)	25.5 (5.09)
Maternal Pre-Pregnancy BMI	26.0 (6.07)	26.11 (6.1)	26.1 (6.26)	25.9 (6.41)	26.4 (6.52)
Missing BMI	7	7	7	7	7
Partnership Status					
Not Partnered	200 (76.9)	201 (75.6)	183 (75.6)	155 (73.8)	157 (75.1)
Partnered	60 (23.1)	65 (24.4)	59 (24.4)	55 (26.2)	52 (24.9)
Parity					
Nulliparous	126 (48.5)	131 (49.2)	121 (50)	106 (50.5)	104 (49.8)
Multiparous	134 (51.5)	135 (50.8)	121 (50)	104 (49.5)	105 (50.2)
Receipt of Public Assistance					
No	155 (59.6)	159 (59.8)	147 (60.7)	124 (59)	125 (59.8)
Yes	105 (40.4)	107 (40.2)	95 (39.3)	86 (41)	84 (40.2)
Education Level					
Less than High School	101 (38.8)	101 (38)	92 (38)	77 (36.7)	80 (38.3)
More than High School	159 (61.2)	165 (62)	150 (62)	133 (63.3)	129 (61.7)

^a^TAPAS ancillary study

Cord PBDEs were moderately to strongly correlated with each other (Pearson’s R = 0.62–0.87), however, they were only very weakly correlated with year 7 PBDEs (Supplemental Fig. S1). BDE-47 was the most prevalent congener, detected in 80% of samples, while BDE-153 was the least, detected in 36% of samples (Table 2).

**Table 2:** Imputed and complete case PBDE concentrations in cord plasma (*n* = 289) and in plasma at year 7 (*n* = 194)

Cord Plasma	Wet weight (ng/mL)					Lipid Weight (ng/g lipid)		
	LOD Range, ng/ml	% > LOD	Imputed Geometric Mean (GSD)	Complete Case Geometric Mean (GSD)	80th %tile	Imputed Geometric Mean (GSD)	Complete Case Geometric Mean (GSD)	80th %tile
BDE-47	4.8–15	79.9	30.3 (3.02)	41.7 (2.72)	78.6	14.0 (2.96)	19.1 (2.66)	36.9
BDE-99	2.9–8.7	49.8	8.77 (2.49)	16.3 (2.51)	16.8	3.74 (2.56)	7.11 (2.48)	7.98
BDE-100	2.0–8.7	40.5	6.62 (2.30)	13.5 (2.31)	10.5	2.89 (2.32)	5.64 (2.33)	5.53
BDE-153	2.0–8.7	36.0	5.73 (1.96)	10.7 (2.04)	8.67	2.65 (1.82)	4.22 (1.95)	3.79
ΣPBDE^a^	–	–	54.7 (2.53)^a^	163 (2.36)	112	24.9 (2.49)	62.9 (2.54)	56
Year 7 Plasma								
BDE-47	6.9–32.3	97.4	89.3 (2.99)	95.1 (2.82)	205	23.0 (2.94)	24.3 (2.83)	53.7
BDE-99	4.2–17.0	79.9	22.9 (3.02)	31.4 (2.73)	61.7	5.81 (3.05)	7.92 (2.78)	14.8
BDE-100	2.9–13.5	91.2	20.4 (2.73)	23.8 (2.47)	47.3	5.24 (2.71)	6.04 (2.48)	11.1
BDE-153	2.9–13.5	93.8	24.6 (2.52)	27.6 (2.29)	52.9	6.34 (2.46)	7.05 (2.26)	13.3
ΣPBDE	–	–	170 (2.65)	224 (2.4)	382	43.6 (2.62)	56.7 (2.44)	98.1

^a^46 participants had detectable levels of all PBDEs in cord blood

PBDEs were not associated with overall child BMIz from ages 5 to 14 years in fully adjusted models (Table 3). However, in minimally adjusted models controlling for age, age^2^, and sex (Model 1), concentrations of BDE-47 ≥ 78.6 ng/mL were associated with a BMIz 0.38 (− 0.69, − 0.06) lower than those with low BDE-47 (*p* = 0.02). Furthermore, BDE-153 ≥ 8.67 ng/mL was associated with a BMIz 0.35 (− 0.67, − 0.03) lower than children with BDE-153 < 8.67 ng/ml (*p* = 0.03). Similarly, children with Σ PBDEs ≥ 112 ng/mL in cord plasma, had a BMIz 0.35 (− 0.67, − 0.03) lower than children with Σ PBDEs < 112 ng/mL. Nevertheless, when models were fully adjusted for covariates, these associations were attenuated. Associations did not change when inverse probability weights were included (Model 3), when PBDEs were lipid standardized (Model 4), nor when models were adjusted for PBDEs measured at the year 7 visit (Model 5) (Supplemental Table S2). No associations between cord concentrations of PBDEs and BMIz were observed when PBDEs were modeled continuously (Supplemental Table S3). Finally, altering the categorization of “high” PBDEs to ≥ the 65th or 90th percentile did not meaningfully alter our findings (Supplemental Table S4).

**Table 3:** Associations between high cord plasma PBDE measures (ng/mL) and average overall child BMI Z-scores from age 5–14 years

PBDE	80th Percentile	Beta Coefficient (95% Confidence Interval)^ad^	
		Model 1^b^	Model 2^c^
BDE-47	≥78.6 ng/mL	−0.38 (− 0.69, − 0.06)**	−0.24 (− 0.55, 0.07)
BDE-99	≥16.8 ng/mL	− 0.29 (− 0.61, 0.02)*	− 0.06 (− 0.37, 0.26)
BDE-100	≥10.5 ng/mL	− 0.22 (− 0.54, 0.1)	− 0.10 (− 0.42, 0.21)
BDE-153	≥8.67 ng/mL	− 0.35 (− 0.67, − 0.03)**	−0.13 (− 0.47, 0.22)
ΣPBDE	≥112 ng/mL	− 0.35 (− 0.67, − 0.03)**	− 0.20 (− 0.52, 0.11)

^a^Beta coefficients are interpreted as the change in BMI Z-score when each PBDE is increased from low (< 80th percentile) to high (≥ 80th percentile) across 10 multiply imputed datasets

^b^Model 1: Minimally adjusted for age and age^2^ (centered at age 5) and sex

^c^Model 2: Fully adjusted for maternal parity (primiparous/multiparous), age at birth, ethnicity (Dominican/African American), receipt of public assistance (yes/no), completed high school (yes/no), partnership status (partnered/single), and linear and quadratic child age at visit (centered at age 5) and child sex

^d^*P*-value Thresholds: ***P ≤ 0.001; **P ≤ 0.05, *P ≤ 0.1

We used quantile g-computation to investigate associations of the total cord plasma PBDE burden and child BMIz. There was no effect of the total PBDE mixture on child BMIz (Ψ = − 0.09 (− 0.25, 0.07), *p* = 0.27). BDE-99 had the greatest estimated weight to the exposure mixture (mean ± standard deviation across 10 multiply imputed datasets = 0.88 ± 0.17), followed by BDE-100 (− 0.85 ± 0.15), BDE-153 (− 0.05 ± 0.26), and BDE-47 (0.01 ± 0.13). However, as the main effect was not significant, these weights should be interpreted with caution.

We next examined models including interactions between each PBDE and child age + age^2^ to determine the associations of PBDEs with child adiposity over time (Table 4). The categorical PBDE*age and categorical PBDE*age^2^ interaction effect estimates describe how the differences in BMIz from low (80th percentile) to high (≥ 80th percentile) exposure change with age. Effect estimates for the linear PBDE*age term and the PBDE*age^2^ term were not significantly different between individuals with high PBDEs and low PBDEs for any congener or the PBDE total. Predicted changes in BMIz over time by PBDE category can be visualized in Supplemental Fig. S2. When BMI was specified as the outcome in place of BMIz, the shape of the trajectories by high and low PBDEs differed (Supplemental Fig. S3); however, the overall results were similar to models of BMIz (Supplemental Table S5). No differences were observed between BMI trajectories for prenatal BDE-47, − 99, or − 153 concentrations; however, a significant interaction between child age and BDE-100 was detected with individuals with high BDE-100 levels experiencing lower BMI over time than those with low BDE-100 (β = − 0.42 (− 0.82, − 0.03); *p* = 0.03). This is similar to models including BMIz, where borderline significant interactions were observed between BDE-100 and age (β = − 0.1 (− 0.2, 0.01) *p* = 0.07) and age^2^ (β = 0.02 (0, 0.03); *p* = 0.06). Results did not change when PBDEs were dichotomized at the 65th or 90th percentiles (Supplemental Table S6), inverse probability weights were included (Model 3), when PBDEs were lipid adjusted (Model 4), nor when models were adjusted for PBDEs measured at the year 7 visit (Model 5) (Supplemental Table S7).

**Table 4:** Associations between high cord plasma PBDE concentrations and trajectories of child BMI z-score from 5 to 14 years

Predictor Variables	Beta Coefficient (95% Confidence Interval)^ab^	
	Model 1^b^	Model 2^c^
BDE-47		
BDE-47 ≥78 ng/mL	−0.36 (− 0.69, − 0.02)**	−0.21 (− 0.54, 0.12)
Centered Age	0.12 (0.07, 0.16)***	0.12 (0.07, 0.16)***
Centered Age^2	− 0.01 (− 0.02, − 0.01)***	−0.01 (− 0.02, − 0.01)***
BDE-47*Centered Age	0.02 (− 0.09, 0.12)	0.02 (− 0.09, 0.12)
BDE-47*Centered Age^2	− 0.01 (− 0.02, 0.01)	− 0.01 (− 0.02, 0.01)
BDE-99		
BDE-99 ≥16.8 ng/mL	− 0.22 (− 0.56, 0.12)	0.02 (− 0.32, 0.36)
Centered Age	0.13 (0.08, 0.17)***	0.13 (0.08, 0.17)***
Centered Age^2	−0.01 (− 0.02, − 0.01)***	−0.01 (− 0.02, − 0.01)***
BDE-99*Centered Age	−0.04 (− 0.14, 0.07)	−0.04 (− 0.15, 0.07)
BDE-99*Centered Age^2	0.003 (− 0.01, 0.02)	0 (− 0.01, 0.02)
BDE-100		
BDE-100 ≥10.5 ng/mL	−0.15 (− 0.48, 0.19)	−0.03 (− 0.36, 0.31)
Centered Age	0.14 (0.09, 0.19) ***	0.14 (0.09, 0.19) ***
Centered Age^2	−0.02 (− 0.02, − 0.01) ***	−0.02 (− 0.02, − 0.01) ***
BDE-100*Centered Age	−0.1 (− 0.2, 0.01)*	−0.1 (− 0.2, 0.01)*
BDE-100*Centered Age^2	0.02 (0, 0.03)*	0.02 (0, 0.03)*
BDE-153		
BDE-153 ≥8.67 ng/mL	−0.37 (− 0.71, − 0.03)**	−0.16 (− 0.52, 0.21)
Centered Age	0.13 (0.08, 0.17)***	0.13 (0.08, 0.17) ***
Centered Age^2	−0.02 (− 0.02, − 0.01)***	−0.02 (− 0.02, − 0.01)***
BDE-153*Centered Age	−0.05 (− 0.15, 0.06)	−0.05 (− 0.15, 0.06)
BDE-153*Centered Age^2	0.01 (0, 0.03)	0.01 (0, 0.03)
ΣPBDE		
Σ PBDE ≥ 112 ng/mL	−0.33 (− 0.67, 0.01)*	−0.18 (− 0.52, 0.16)
Centered Age	0.12 (0.07, 0.16)***	0.12 (0.07, 0.16)***
Centered Age^2	−0.01 (− 0.02, − 0.01***	−0.01 (− 0.02, − 0.01)***
Σ PBDE*Centered Age	0.02 (− 0.09, 0.12)	0.01 (− 0.09, 0.12)
Σ PBDE*Centered Age^2	0.004 (− 0.02, 0.01)	0 (− 0.02, 0.01)

^a^Beta coefficients are interpreted as the change in BMI Z-score when each PBDE is increased from low (< 80th percentile) to high (>80th percentile) when age is increased by one year

^b^Model 1: Minimally adjusted for age and age^2^ (centered at age 5) and sex

^c^Model 2: Fully adjusted for maternal parity (primiparous/multiparous), age at birth, ethnicity (Dominican/African American), receipt of public assistance (yes/no), completed high school (yes/no), partnership status (partnered/single), and linear and quadratic child age at visit (centered at age 5) and child sex

^b^*P*-value Thresholds: ***P ≤ 0.001; **P ≤ 0.05, *P ≤ 0.1

Associations between cord PBDE measurements and child BMIz did not differ by child sex nor by ethnicity (Table 5).

**Table 5:** Associations between high PBDE exposure and child BMI z-score stratified by child sex and by child race/ethnicity

	Beta Coefficient (95% Confidence Interval)^ab^					
	Sex-Stratified			Ethnicity-Stratified		
PBDE	Male	Female	Interaction *P* Value	Dominican	African American	Interaction *P* Value
BDE-47 > 36.9 ng/mL	−0.34 (− 0.76, 0.08)	−0.17 (− 0.63, 0.3)	0.41	−0.26 (− 0.7, 0.18)	−0.04 (− 0.5, 0.43)	0.14
BDE-99 > 7.98 ng/mL	−0.28 (− 0.73, 0.16)	0.1 (− 0.36, 0.56)	0.24	0.003 (− 0.47, 0.48)	0.05 (− 0.39, 0.5)	0.73
BDE-100 > 5.53 ng/mL	−0.23 (− 0.66, 0.2)	−0.06 (− 0.52, 0.4)	0.47	−0.12 (− 0.55, 0.32)	0.05 (− 0.41, 0.51)	0.51
BDE-153 > 3.79 ng/mL	−0.28 (− 0.74, 0.17)	−0.1 (− 0.63, 0.44)	0.47	−0.18 (− 0.65, 0.3)	0.09 (− 0.42, 0.61)	0.46
ΣPBDE > 56 ng/mL	−0.33 (− 0.76, 0.09)	−0.14 (− 0.61, 0.34)	0.38	−0.18 (− 0.62, 0.26)	−0.07 (− 0.54, 0.39)	0.2

^a^Beta coefficients are interpreted as the change in BMI Z-score when each PBDE is increased from low (< 80th percentile) to high (>80th percentile) in models adjusted for maternal parity (primiparous/multiparous), age at birth, ethnicity (Dominican/African American), receipt of public assistance (yes/no), completed high school (yes/no), partnership status (partnered/single), and linear and quadratic child age at visit (centered at age 5) and child sex with IPW

*P*-value Thresholds: ***P ≤ 0.001; **P ≤ 0.05, *P ≤ 0.1

## Discussion

PBDEs are a class of legacy flame retardants that were extensively used in consumer products between 1975 and 2004 to satisfy fire safety regulatory requirements. As PBDEs are not chemically bonded to the product matrix, they are able to leach from their original product into the environment, resulting in exposures to pregnant women [27]. In our analysis of 289 Dominican and African American mother-child pairs, cord plasma measures of PBDEs were not associated with child BMIz or the change in BMIz over time. We also found no evidence of effect modification by child sex or by race/ethnicity.

Prenatal development is highly susceptible to environmental insult and environmental compounds have been identified to alter adipocyte lineage during embryonic development [40]. PBDEs are putative thyroid hormone disruptors, with recognized impacts on neurodevelopment [41]. Experimental in vitro and in vivo studies suggest that PBDE exposures may lead to altered signaling surrounding adipogenesis and weight control. For instance, perinatal exposure to BDE-99 increased rat pup body weight and liver markers of oxidative stress [12] and perinatal exposure to the PBDE mixture, DE-71, increased rat pup body and liver weights [13]. These changes were accompanied by alterations in liver and adipose tissue metabolism. In a study of perinatal exposure to BDE-47, liver gene expression changes were observed in biological pathways related to carbohydrate, lipid, fatty acid and steroid metabolism [15]. In vitro, BDE-47 induced adipocyte differentiation of 3T3-L1 cells and increased adipokine gene expression [17, 18].

Despite the evidence from experimental animal studies, analyses examining prenatal PBDE exposures and childhood adiposity in human populations are mixed. Similar to our findings, an examination of maternal serum PBDEs with childhood BMIz at 7 years found no associations between prenatal PBDE concentrations and childhood BMIz [20]. However, an analysis of 318 mother-child pairs from Cincinnati, OH, found that increasing concentrations of BDE-153 in second trimester maternal serum were associated with lower childhood BMI z-score, waist circumference, and percent body fat from ages 1–8 years [19]. Similarly, BDE-153 was negatively associated with child BMI z-score at age 7 in 224 mother-child pairs from the Salinas Valley, CA [42]. In contrast to our findings, several studies have also observed sex-specific associations between prenatal PBDE levels and childhood adiposity. For example, in the same study population from the Salinas Valley, BDE-153 was positively associated with BMIz in boys, but negatively associated with BMIz in girls [42]. In contrast, in a study of lactational PBDE exposures and childhood anthropometrics at 36 months, researchers observed positive associations with BDE-153 and weight-for-height z-scores in girls, but negative associations in boys [43]. Inconsistent findings between studies may be due to variability in timing of outcome measurements, different periods of PBDE exposure measurements (i.e., before or after phase-out), geographical location, or different ethnic/racial backgrounds of participants. However, though PBDE concentrations differed between Dominican and African American participants [29], we observed no evidence of effect modification by race/ethnicity. The levels of cord PBDE concentrations measured in the present analysis are consistent with other studies examining PBDEs in umbilical cord and maternal blood in the United States during a similar time period [22, 44–48].

This study has several strengths, including the focus on minority populations, long-term follow-up across four study visits, investigation of overall BMIz and BMIz trajectories, use of an advanced mixtures approach, and the ability to control for several key confounders and covariates. We were further able to explore the influence of postnatal exposures by adjusting for PBDEs measured at age 7. However, like all studies, this analysis is subject to several limitations. The large number of PBDE values below the limit of detection may have impacted our findings; however, our analyses included both categorical and continuous PBDE exposures with multiple imputations for missing values. Additionally, we adjusted our models for cord lipid contents as confounders; however, it is possible that cord lipids may be a mediator between PBDEs and adiposity. In this case, adjusting for lipid content would lead to attenuated findings. We thus included models both adjusted and unadjusted for lipids. We also note that the impact of lipid content on PBDE measurement was likely quite high, given the extremely high octanol: water partitioning coefficient for PBDEE congeners and that serum lipids are the determinant of blood: adipose partitioning in humans [49, 50], suggesting that plasma lipids are a primary determinant of PBDE measurements. Next, we are likely underpowered to detect sex-specific or ethnicity-specific effects, suggesting that follow-up in larger populations is necessary. Furthermore, while some loss-to-follow-up occurred as is typical of longitudinal cohort studies; we addressed potential biases by including inverse probability weights in our sensitivity analyses. Finally, we were confined to investigating associations with child BMIz, which may not be the optimal indicator of childhood adiposity. Future studies would benefit from a more precise measure of childhood total adiposity and where in the body the adipose tissue is located, such as magnetic resonance imaging.

In conclusion, cord plasma PBDE levels were not associated with childhood BMI z-scores from 5 to 14 years in this sample. This research contributes to the weight of human population evidence aiming to elucidate whether relationships exist between PBDE exposures and childhood adiposity. Future studies should examine these associations using external measures of exposure, such as house dust or passive sampling silicon wristbands, to address issues of reverse causality. Research in larger, more diverse, cohorts would be further crucial to confirm these findings.

## Supplementary Information

**Additional file 1: Supplemental Fig. S1.** Mean cord PBDE Pearson’s correlations across 10 multiply imputed datasets. The gradient indicates the strength of the correlation. **Supplemental Fig. S2.** Predicted BMI Z-score growth trajectories from age 5-14 years for children with high (blue, >80th percentile) and low (red) PBDEs. **Supplemental Table S1.** Summary of follow-up information for the 289 individuals included in the present analysis and the full population of 541 individuals with a BMI measurement between ages 5 and 14. **Supplemental Table S2.** Sensitivity analyses for associations between cord plasma PBDE measures and overall child BMI Z-scores from age 5–14 years. **Supplemental Table S3.** Associations between continuous cord plasma PBDE measures and overall child BMI Z-scores from age 5–14 years. **Supplemental Table S4.** Sensitivity analyses for associations between cord plasma PBDE dichotomized at the 65th and 90th percentiles and overall child BMI Z-scores from age 5–14 years. **Supplemental Table S5.** Sensitivity analyses for associations between cord plasma PBDEs and trajectories of child BMI from 5 to 14 years. **Supplemental Table S6.** Sensitivity analyses for associations between cord plasma PBDEs dichotomized at the 65th and 90th percentiles and trajectories of child BMI z-score from 5 to 14 years. **Supplemental Table S7.** Sensitivity analyses for associations between cord plasma PBDEs and trajectories of child BMI z-score from 5 to 14 years.

## Data Availability

The datasets analyzed during the current study are not publicly available due to participant confidentiality but are available from the corresponding author on reasonable request following appropriate human subjects training and IRB approval.

## References

[1] National Center for Health Statistics (US) (2017). Health, United States, 2016: with Chartbook on long-term trends in health [internet].

[2] Ogden CL, Carroll MD, Kit BK, Flegal KM (2012). Prevalence of obesity and trends in body mass index among US children and adolescents, 1999-2010. JAMA..

[3] Thayer KA, Heindel JJ, Bucher JR, Gallo MA (2012). Role of environmental Chemicals in Diabetes and Obesity: a National Toxicology Program Workshop Review. Environ Health Perspect.

[4] Braun JM (2017). Early life exposure to endocrine disrupting chemicals and childhood obesity and neurodevelopment. Nat Rev Endocrinol.

[5] Ravelli AC, van Der Meulen JH, Osmond C, Barker DJ, Bleker OP (1999). Obesity at the age of 50 y in men and women exposed to famine prenatally. Am J Clin Nutr.

[6] Yang Z, Zhao W, Zhang X, Mu R, Zhai Y, Kong L (2008). Impact of famine during pregnancy and infancy on health in adulthood. Obes Rev.

[7] Hites RA (2004). Polybrominated Diphenyl ethers in the environment and in people: a meta-analysis of concentrations. Environ Sci Technol.

[8] Harrad S, Diamond M (2006). New directions: exposure to polybrominated diphenyl ethers (PBDEs) and polychlorinated biphenyls (PCBs): current and future scenarios. Atmos Environ.

[9] Csiszar SA, Diamond ML, Daggupaty SM (2014). The magnitude and spatial range of current-use urban PCB and PBDE emissions estimated using a coupled multimedia and air transport model. Environ Sci Technol.

[10] Grün F, Blumberg B (2009). Endocrine disrupters as obesogens. Mol Cell Endocrinol.

[11] Taylor KW, Novak RF, Anderson HA, Birnbaum LS, Blystone C, DeVito M (2013). Evaluation of the association between persistent organic pollutants (POPs) and diabetes in epidemiological studies: a National Toxicology Program Workshop Review. Environ Health Perspect.

[12] Blanco J, Mulero M, Domingo JL, Sanchez DJ (2014). Perinatal exposure to BDE-99 causes decreased protein levels of Cyclin D1 via GSK3β activation and increased ROS production in rat pup livers. Toxicol Sci.

[13] Bondy GS, Lefebvre DE, Aziz S, Cherry W, Coady L, MacLellan E (2013). Toxicologic and immunologic effects of perinatal exposure to the brominated diphenyl ether (BDE) mixture DE-71 in the Sprague-Dawley rat. Environ Toxicol.

[14] Gee JR, Moser VC (2008). Acute postnatal exposure to brominated diphenylether 47 delays neuromotor ontogeny and alters motor activity in mice. Neurotoxicol Teratol.

[15] Suvorov A, Battista M-C, Takser L (2009). Perinatal exposure to low-dose 2,2′,4,4′-tetrabromodiphenyl ether affects growth in rat offspring: what is the role of IGF-1?. Toxicology..

[16] Suvorov A, Takser L (2010). Global gene expression analysis in the livers of rat offspring perinatally exposed to low doses of 2,2′,4,4′-tetrabromodiphenyl ether. Environ Health Perspect.

[17] Bastos Sales L, Kamstra JH, Cenijn PH, van Rijt LS, Hamers T, Legler J (2013). Effects of endocrine disrupting chemicals on in vitro global DNA methylation and adipocyte differentiation. Toxicol in Vitro.

[18] Kamstra JH, Hruba E, Blumberg B, Janesick A, Mandrup S, Hamers T (2014). Transcriptional and epigenetic mechanisms underlying enhanced in vitro adipocyte differentiation by the brominated flame retardant BDE-47. Environ Sci Technol.

[19] Vuong AM, Braun JM, Sjödin A, Webster GM, Yolton K, Lanphear BP (2016). Prenatal Polybrominated Diphenyl ether exposure and body mass index in children up to 8 years of age. Environ Health Perspect.

[20] Agay-Shay K, Martinez D, Valvi D, Garcia-Esteban R, Basagaña X, Robinson O (2015). Exposure to endocrine-disrupting chemicals during pregnancy and weight at 7 years of age: a multi-pollutant approach. Environ Health Perspect.

[21] Zota AR, Adamkiewicz G, Morello-Frosch RA (2010). Are PBDEs an environmental equity concern? Exposure disparities by socioeconomic status. Environ Sci Technol.

[22] Horton MK, Bousleiman S, Jones R, Sjodin A, Liu X, Whyatt R (2013). Predictors of serum concentrations of polybrominated flame retardants among healthy pregnant women in an urban environment: a cross-sectional study. Environ Health.

[23] Perera FP, Rauh V, Whyatt RM, Tsai W-Y, Tang D, Diaz D (2006). Effect of prenatal exposure to airborne polycyclic aromatic hydrocarbons on neurodevelopment in the first 3 years of life among Inner-City children. Environ Health Perspect.

[24] Perera FP, Illman SM, Kinney PL, Whyatt RM, Kelvin EA, Shepard P (2002). The challenge of preventing environmentally related disease in young children: community-based research in new York City. Environ Health Perspect.

[25] Rauh VA, Whyatt RM, Garfinkel R, Andrews H, Hoepner L, Reyes A (2004). Developmental effects of exposure to environmental tobacco smoke and material hardship among inner-city children. Neurotoxicol Teratol.

[26] Rundle AG, Gallagher D, Herbstman JB, Goldsmith J, Holmes D, Hassoun A (2019). Prenatal exposure to airborne polycyclic aromatic hydrocarbons and childhood growth trajectories from age 5-14 years. Environ Res.

[27] Talsness CE (2008). Overview of toxicological aspects of polybrominated diphenyl ethers: a flame-retardant additive in several consumer products. Environ Res.

[28] Sjödin A, Jones RS, Lapeza CR, Focant J-F, McGahee EE, Patterson DG (2004). Semiautomated high-throughput extraction and cleanup method for the measurement of Polybrominated Diphenyl ethers, Polybrominated biphenyls, and polychlorinated biphenyls in human serum. Anal Chem.

[29] Cowell WJ, Sjödin A, Jones R, Wang Y, Wang S, Herbstman JB (2018). Determinants of prenatal exposure to polybrominated diphenyl ethers (PBDEs) among urban, minority infants born between 1998 and 2006. Environ Pollut.

[30] Baccarelli A, Pfeiffer R, Consonni D, Pesatori AC, Bonzini M, Patterson DG (2005). Handling of dioxin measurement data in the presence of non-detectable values: overview of available methods and their application in the Seveso chloracne study. Chemosphere..

[31] Van Buuren S, Groothuis-Oudshoorn K (2011). Mice: multivariate imputation by chained equations in R. J Stat Softw.

[32] Kontopantelis E, White IR, Sperrin M, Buchan I (2017). Outcome-sensitive multiple imputation: a simulation study. BMC Med Res Methodol.

[33] Berkey CS, Colditz GA (2007). Adiposity in adolescents: change in actual BMI works better than change in BMI z score for longitudinal studies. Ann Epidemiol.

[34] Paluch RA, Epstein LH, Roemmich JN (2007). Comparison of methods to evaluate changes in relative body mass index in pediatric weight control. Am J Hum Biol Off J Hum Biol Counc.

[35] Cole TJ, Faith MS, Pietrobelli A, Heo M (2005). What is the best measure of adiposity change in growing children: BMI, BMI %, BMI z-score or BMI centile?. Eur J Clin Nutr.

[36] Keil AP, Buckley JP, O’Brien KM, Ferguson KK, Zhao S, White AJ (2020). A Quantile-based g-computation approach to addressing the effects of exposure mixtures. Environ Health Perspect.

[37] Rubin DB. Multiple Imputation for Nonresponse in Surveys. New York: John Wiley & Sons, Inc.; 1987.

[38] R Core Team (2018). R: a language and environment for statistical computing.

[39] van der Wal WM, Geskus RB (2011). Ipw: an R package for inverse probability weighting. J Stat Softw.

[40] Heindel JJ, Blumberg B, Cave M, Machtinger R, Mantovani A, Mendez MA (2017). Metabolism disrupting chemicals and metabolic disorders. Reprod Toxicol.

[41] Costa LG, de Laat R, Tagliaferri S, Pellacani C (2014). A mechanistic view of polybrominated diphenyl ether (PBDE) developmental neurotoxicity. Toxicol Lett.

[42] Erkin-Cakmak A, Harley KG, Chevrier J, Bradman A, Kogut K, Huen K (2015). In utero and childhood Polybrominated Diphenyl ether exposures and body mass at age 7 years: the CHAMACOS study. Environ Health Perspect.

[43] Hoffman K, Mendez M, Siega-Riz AM, Herring AH, Sjödin A, Daniels JL (2016). Lactational exposure to Polybrominated Diphenyl ethers and its relation to early childhood anthropometric measurements. Environ Health Perspect.

[44] Stapleton HM, Eagle S, Anthopolos R, Wolkin A, Miranda ML (2011). Associations between Polybrominated Diphenyl ether (PBDE) flame retardants, phenolic metabolites, and thyroid hormones during pregnancy. Environ Health Perspect.

[45] Vuong AM, Webster GM, Romano ME, Braun JM, Zoeller RT, Hoofnagle AN (2015). Maternal Polybrominated Diphenyl ether (PBDE) exposure and thyroid hormones in maternal and cord sera: the HOME study, Cincinnati, USA. Environ Health Perspect.

[46] Herbstman JB, Sjödin A, Apelberg BJ, Witter FR, Patterson DG, Halden RU (2007). Determinants of prenatal exposure to polychlorinated biphenyls (PCBs) and polybrominated diphenyl ethers (PBDEs) in an urban population. Environ Health Perspect.

[47] Castorina R, Bradman A, Sjödin A, Fenster L, Jones RS, Harley KG (2011). Determinants of serum Polybrominated Diphenyl ether (PBDE) levels among pregnant women in the CHAMACOS cohort. Environ Sci Technol.

[48] Frederiksen M, Thomsen C, Frøshaug M, Vorkamp K, Thomsen M, Becher G (2010). Polybrominated diphenyl ethers in paired samples of maternal and umbilical cord blood plasma and associations with house dust in a Danish cohort. Int J Hyg Environ Health.

[49] Yue C, Li LY (2013). Filling the gap: estimating physicochemical properties of the full array of polybrominated diphenyl ethers (PBDEs). Environ Pollut.

[50] Haddad S, Poulin P, Krishnan K (2000). Relative lipid content as the sole mechanistic determinant of the adipose tissue:blood partition coefficients of highly lipophilic organic chemicals. Chemosphere..

