# Supplementary material for: Prenatal exposure to polybrominated diphenyl ethers and BMI Z-scores from 5 to 14 years
Source: Environ Health. 2022 Sep 8;21:82. doi: 10.1186/s12940-022-00893-5 (PMC9454187; doi:10.1186/s12940-022-00893-5)
Supplement: Supplementary file 1 — Additional file 1: Supplemental Fig. S1. Mean cord PBDE Pearson’s correlations across 10 multiply imputed datasets. The gradient indicates the strength of the correlation. Supplemental Fig. S2. Predicted BMI Z-score growth trajectories from age 5-14 years for children with high (blue, >80th percentile) and low (red) PBDEs. Supplemental Table S1. Summary of follow-up information for the 289 individuals included in the present analysis and the full population of 541 individuals with a BMI measurement between ages 5 and 14. Supplemental Table S2. Sensitivity analyses for associations between cord plasma PBDE measures and overall child BMI Z-scores from age 5–14 years. Supplemental Table S3. Associations between continuous cord plasma PBDE measures and overall child BMI Z-scores from age 5–14 years. Supplemental Table S4. Sensitivity analyses for associations between cord plasma PBDE dichotomized at the 65th and 90th percentiles and overall child BMI Z-scores from age 5–14 years. Supplemental Table S5. Sensitivity analyses for associations between cord plasma PBDEs and trajectories of child BMI from 5 to 14 years. Supplemental Table S6. Sensitivity analyses for associations between cord plasma PBDEs dichotomized at the 65th and 90th percentiles and trajectories of child BMI z-score from 5 to 14 years. Supplemental Table S7. Sensitivity analyses for associations between cord plasma PBDEs and trajectories of child BMI z-score from 5 to 14 years. [file 12940_2022_893_MOESM1_ESM.pdf]

Supplemental Material

Prenatal Exposure to Polybrominated Diphenyl Ethers and BMI Z-Scores from 5 to 14 Years

Allison Kupsco[1], Andreas Sjödin[2], Whitney Cowell[3], Richard Jones[2], Sharon Oberfield[4], Shuang Wang[5], Lori A Hoepner[1,6], Dympna Gallagher[7], Andrea A Baccarelli[1], Jeff Goldsmith[5], Andrew G Rundle[8], Julie B Herbstman[1]

[1]Department of Environmental Health Sciences, Mailman School of Public Health, Columbia University, New York, NY

[2]Division of Laboratory Sciences, National Center for Environmental Health, Centers for Disease Control and Prevention, Atlanta, GA

[3]Icahn School of Medicine at Mount Sinai, New York, NY USA

[4]Division of Pediatric Endocrinology, Diabetes and Metabolism, Department of Pediatrics, New York-Presbyterian Morgan Stanley Children's Hospital, New York, New York.

[5]Department of Biostatistics, Mailman School of Public Health, Columbia University, New York, NY

[6]Department of Environmental and Occupational Health Sciences, School of Public Health, SUNY Downstate Health Sciences University, Brooklyn, NY

[7]Nutrition Obesity Research Center, Columbia University Medical Center, New York, NY

[8]Department of Epidemiology, Mailman School of Public Health, Columbia University, New York, NY

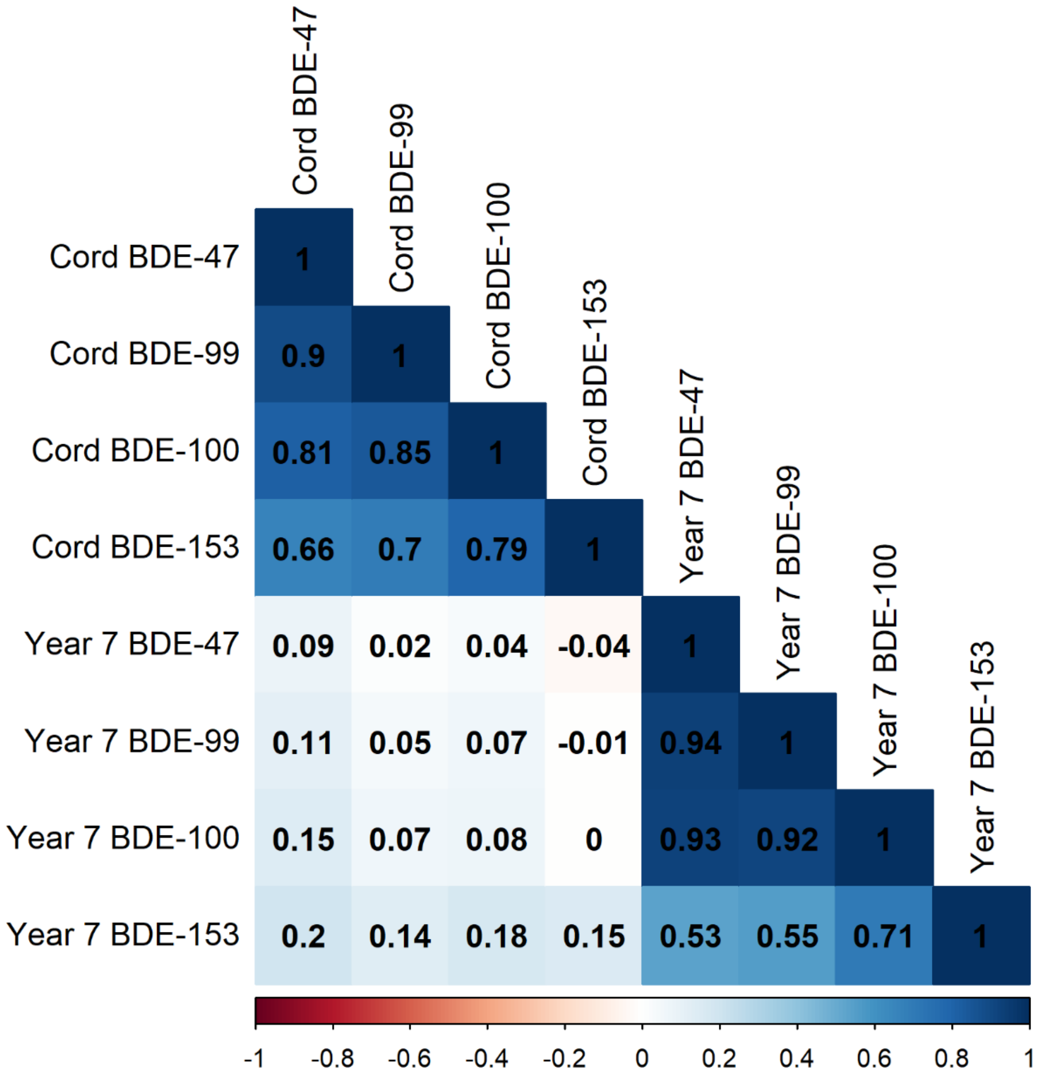

Supplemental Figure S1. Mean cord PBDE Pearson's correlations across 10 multiply imputed datasets. The gradient indicates the strength of the correlation.

Supplemental Figure S2. Predicted BMI Z-score growth trajectories from age 5 14 years for children with high (blue, >80th percentile) and low (red) PBDEs.

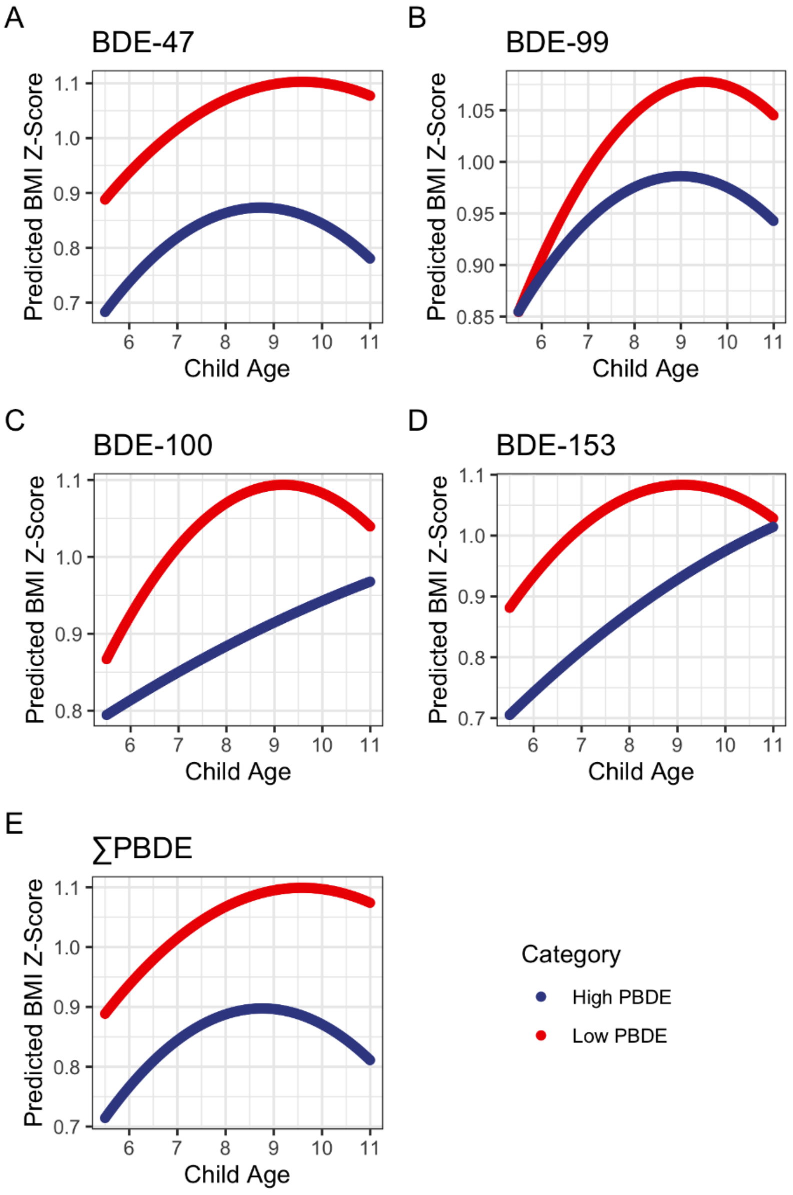

Supplemental Figure S2. Predicted BMI Z score growth trajectories from age 5 14 years for children with high (blue, >80th percentile) and low (red) PBDEs.

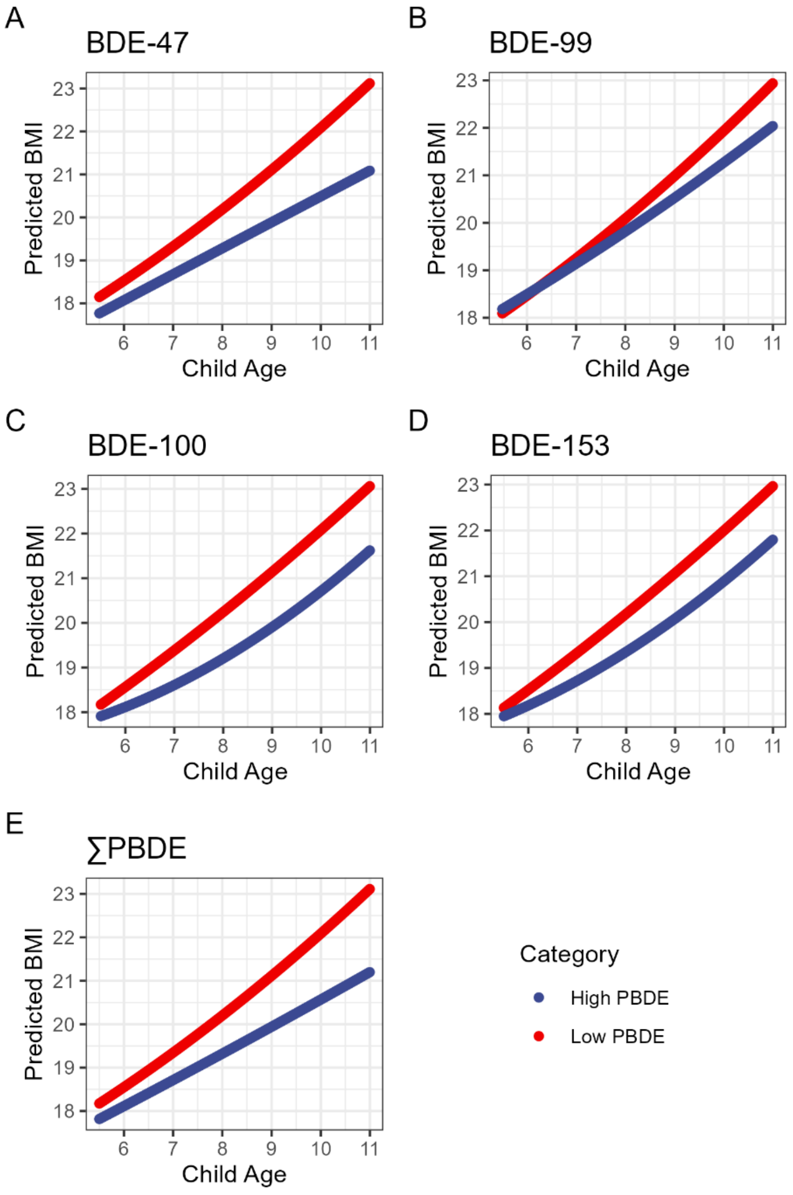

Supplemental Table S1. Summary of follow-up information for the 289 individuals included in the present analysis and the full population of 541 individuals with a BMI measurement between ages 5 and 14.

	Attended[a]						
Only Attended Visit(s)	5	7	9	8 to 14	11	N Study Population	N Full Population
5						8	19
7						2	3
9						0	2
8to14						0	0
11						2	2
5 & 7						21	37
5 & 9						1	1
5 & 8to14						0	0
5 & 11						1	1
7 & 9						1	2
7 & 8to14						0	0
7 & 11						0	0
9 & 8to14						0	0
9 & 11						2	2
8to14 & 11						0	2
5 & 7 & 9						24	59
5 & 7 & 8to14						1	4
5 & 7 & 11						0	2
5 & 9 & 8to14						0	1
5 & 9 & 11						1	1
5 & 8to14 & 11						2	3
7 & 9 & 8to14						4	6
7 & 9 & 11						0	2
7 & 8to14 & 11						1	4
9 & 8to14 & 11						1	3
5 & 7 & 9 & 8to14						17	46
5 & 7 & 9 & 11						17	25
5 & 7 & 8to14 & 11						9	13
5 & 9 & 8to14 & 11						5	7
7 & 9 & 8to14 & 11						16	35
5 & 7 & 9 & 8to14 & 11						153	259

[a]Shaded cells indicate that each visit was attended by the number of participants indicated in N Study Population and N Full Population

Supplemental Table S2. Sensitivity analyses for associations between cord plasma PBDE measures and overall child BMI Z-scores from age 5–14 years.

		Beta Coefficient (95% Confidence Interval)[a,e]		
PBDE	80th %	Model 3[b]	Model 4[c]	Model 5[d]
BDE-47	≥ 78.6 ng/mL	-0.24 (-0.56, 0.07)	-0.18 (-0.49, 0.12)	-0.18 (-0.56, 0.19)
BDE-99	≥16.8 ng/mL	-0.05 (-0.37, 0.27)	0.001 (-0.31, 0.31)	0.01 (-0.36, 0.38)
BDE-100	≥10.5 ng/mL	-0.12 (-0.43, 0.19)	-0.13 (-0.44, 0.19)	0.01 (-0.37, 0.39)
BDE-153	≥8.67 ng/mL	-0.13 (-0.48, 0.22)	-0.19 (-0.52, 0.13)	-0.23 (-0.66, 0.19)
ΣPBDE	≥112 ng/mL	-0.2 (-0.51, 0.12)	-0.13 (-0.44, 0.17)	-0.16 (-0.54, 0.22)

[a]Beta coefficients are interpreted as the change in BMI Z-score when each PBDE is increased from low (< 80th percentile) to high (>80th percentile)
[b]Model 3: Fully adjusted for maternal parity (primiparous/multiparous), age at birth, ethnicity (Dominican/African American), receipt of public assistance (yes/no), completed high school (yes/no), partnership status (partnered/single), and linear and quadratic child age at visit (centered at age 5) and child sex with inverse probability weighting for loss to follow-up
[c]Model 4: Fully adjusted models using lipid adjusted PBDEs
[d]Model 5: Additionally adjusted for PBDEs measured at year 7 (n=194)
[e]P-value Thresholds: ***P≤0.001; **P≤0.05, *P≤0.1

Supplemental Table S3. Associations between continuous cord plasma PBDE measures and overall child BMI Z-scores from age 5–14 years.

	Beta Coefficient (95% Confidence Interval)[a,g]				
	Primary Analyses		Secondary Analyses		
PBDE	Model 1[b]	Model 2[c]	Model 3[d]	Model 4[e]	Model 5[f]
BDE-47	-0.06 (-0.18, 0.05)	-0.02 (-0.13, 0.1)	-0.02 (-0.14, 0.1)	-0.02 (-0.14, 0.09)	0.02 (-0.12, 0.15)
BDE-99	-0.11 (-0.25, 0.03)	-0.02 (-0.17, 0.12)	-0.02 (-0.16, 0.13)	-0.03 (-0.16, 0.11)	0.03 (-0.14, 0.2)
BDE-100	-0.14 (-0.3, 0.01)*	-0.07 (-0.23, 0.09)	-0.07 (-0.24, 0.09)	-0.07 (-0.22, 0.09)	-0.03 (-0.22, 0.15)
BDE-153	-0.2 (-0.39, 0)**	-0.08 (-0.31, 0.14)	-0.09 (-0.31, 0.14)	-0.09 (-0.32, 0.13)	0.05 (-0.2, 0.29)
ΣPBDE	-0.1 (-0.24, 0.04)	-0.04 (-0.18, 0.1)	-0.2 (-0.51, 0.12)	-0.04 (-0.17, 0.1)	0.02 (-0.14, 0.18)

[a]Beta coefficients are interpreted as the change in BMI Z-score per natural log increase in each PBDE across 10 multiply imputed datasets.
[b]Model 1: Minimally adjusted for age and age^2 (centered at age 5) and sex
[c]Model 2: Fully adjusted for maternal parity (primiparous/multiparous), age at birth, ethnicity (Dominican/African American), receipt of public assistance (yes/no), completed high school (yes/no), partnership status (partnered/single), and linear and quadratic child age at visit (centered at age 5) and child sex
[d]Model 3: Fully adjusted models with inverse probability weighting for loss to follow-up
[e]Model 4: Fully adjusted models using lipid adjusted PBDEs
[f]Model 5: Additionally adjusted for PBDEs measured at year 7 (n=194)
[g]P-value Thresholds: ***P≤0.001; **P≤0.05, *P≤0.1

Supplemental Table S4. Sensitivity analyses for associations between cord plasma PBDE dichotomized at the 65th and 90th percentiles and overall child BMI Z-scores from age 5–14 years.

		Beta Coefficient (95% Confidence Interval)[a]
PBDE	65th Percentile	Model 2[b]
BDE-47	≥42.1 ng/mL	0 (-0.27, 0.27)
BDE-99	≥8.72 ng/mL	-0.08 (-0.35, 0.2)
BDE-100	≥7 ng/mL	-0.16 (-0.45, 0.13)
BDE-153	≥6.25 ng/mL	-0.17 (-0.48, 0.14)
ΣPBDE	≥64.2 ng/mL	-0.11 (-0.39, 0.16)
	90th Percentile	Model 2[b]
BDE-47	≥139 ng/mL	-0.05 (-0.47, 0.36)
BDE-99	≥35.1 ng/mL	0.03 (-0.4, 0.45)
BDE-100	≥24.7 ng/mL	0.06 (-0.36, 0.48)
BDE-153	≥14.4 ng/mL	0.22 (-0.22, 0.66)
ΣPBDE	≥201 ng/mL	-0.1 (-0.52, 0.32)

[a]Beta coefficients are interpreted as the change in BMI Z-score when each PBDE is increased from low (< 80th percentile) to high (>80th percentile)

[b]Model 2: Fully adjusted for maternal parity (primiparous/multiparous), age at birth, ethnicity (Dominican/African American), receipt of public assistance (yes/no), completed high school (yes/no), partnership status (partnered/single), and linear and quadratic child age at visit (centered at age 5) and child sex with inverse probability weighting for loss to follow-up

Supplemental Table S5. Sensitivity analyses for associations between cord plasma PBDEs and trajectories of child BMI from 5 – 14 years.

	Beta Coefficient (95% Confidence Interval)[ab]
Predictor Variables	Model 2
BDE-47	
BDE-47	-0.32 (-1.53, 0.89)
Centered Age	0.71 (0.54, 0.88)***
Centered Age^2	0.03 (0, 0.06)**
BDE-47*Centered Age	-0.1 (-0.5, 0.29)
BDE-47*Centered Age^2	-0.03 (-0.09, 0.03)
BDE-99	
BDE-99	0.14 (-1.1, 1.39)
Centered Age	0.7 (0.53, 0.88)***
Centered Age^2	0.03 (0, 0.05)**
BDE-99*Centered Age	-0.1 (-0.5, 0.3)
BDE-99*Centered Age^2	-0.01 (-0.07, 0.05)
BDE-100	
BDE-100	-0.06 (-1.27, 1.16)
Centered Age	0.76 (0.59, 0.93)***
Centered Age^2	0.02 (-0.01, 0.05)
BDE-100*Centered Age	-0.42 (-0.82, -0.03)**
BDE-100*Centered Age^2	0.03 (-0.03, 0.09)
BDE-153	
BDE-153	-0.01 (-1.33, 1.3)
Centered Age	0.75 (0.58, 0.92)***
Centered Age^2	0.02 (-0.01, 0.05)
BDE-153*Centered Age	-0.35 (-0.75, 0.05)
BDE-153*Centered Age^2	0.03 (-0.04, 0.09)
ΣPBDE	
ΣPBDE	-0.29 (-1.51, 0.93)
Centered Age	0.71 (0.54, 0.88)*
Centered Age^2	0.03 (0, 0.06)**
ΣPBDE*Centered Age	-0.12 (-0.52, 0.28)
ΣPBDE*Centered Age^2	-0.02 (-0.09, 0.04)

[a]Fully adjusted for maternal parity (primiparous/multiparous), age at birth, ethnicity (Dominican/African American), receipt of public assistance (yes/no), completed high school (yes/no), partnership status (partnered/single), and linear and quadratic child age at visit (centered at age 5) and child sex with inverse probability weighting for loss to follow-up
[b]P-value Thresholds: ***P≤0.001; **P≤0.05, *P≤0.1

Supplemental Table S6. Sensitivity analyses for associations between cord plasma PBDEs dichotomized at the 65th and 90th percentiles and trajectories of child BMI z-score from 5 – 14 years.

	Beta Coefficient (95% Confidence Interval)[ab]	
Predictor Variables	65th Percentile[c]	90th Percentile[d]
BDE-47		
BDE-47	-0.06 (-0.34, 0.23)	0 (-0.45, 0.44)
Centered Age	0.11 (0.06, 0.16)***	0.12 (0.08, 0.17)***
Centered Age^2	-0.01 (-0.02, -0.01)***	-0.01 (-0.02, -0.01)***
BDE-47*Centered Age	0.02 (-0.07, 0.1)	-0.04 (-0.18, 0.1)
BDE-47*Centered Age^2	0 (-0.01, 0.01)	0.01 (-0.02, 0.03)
BDE-99		
BDE-99	-0.14 (-0.43, 0.15)	0.07 (-0.38, 0.52)
Centered Age	0.1 (0.05, 0.15)***	0.12 (0.08, 0.17)***
Centered Age^2	-0.01 (-0.02, 0)**	-0.01 (-0.02, -0.01)***
BDE-99*Centered Age	0.05 (-0.04, 0.13)	-0.05 (-0.19, 0.09)
BDE-99*Centered Age^2	-0.01 (-0.02, 0.01)	0.01 (-0.01, 0.03)
BDE-100		
BDE-100	-0.24 (-0.56, 0.09)	0.13 (-0.31, 0.58)
Centered Age	0.11 (0.06, 0.17)***	0.13 (0.09, 0.17)***
Centered Age^2	-0.01 (-0.02, -0.01)***	-0.02 (-0.02, -0.01)***
BDE-100*Centered Age	0.02 (-0.08, 0.12)	-0.09 (-0.23, 0.04)
BDE-100*Centered Age^2	0 (-0.01, 0.02)	0.01 (-0.01, 0.04)
BDE-153		
BDE-153	-0.23 (-0.58, 0.11)	0.32 (-0.14, 0.79)
Centered Age	0.11 (0.05, 0.16)***	0.13 (0.08, 0.17)***
Centered Age^2	-0.01 (-0.02, 0)**	-0.01 (-0.02, -0.01)***
BDE-153*Centered Age	0.04 (-0.06, 0.14)	-0.06 (-0.21, 0.08)
BDE-153*Centered Age^2	0 (-0.02, 0.01)	0.01 (-0.02, 0.03)
ΣPBDE		
ΣPBDE	-0.17 (-0.46, 0.13)	-0.08 (-0.53, 0.37)
Centered Age	0.11 (0.06, 0.16)***	0.12 (0.08, 0.17)***
Centered Age^2	-0.01 (-0.02, -0.01)***	-0.01 (-0.02, -0.01)***
ΣPBDE*Centered Age	0.01 (-0.08, 0.09)	-0.04 (-0.18, 0.09)
ΣPBDE*Centered Age^2	0 (-0.01, 0.02)	0.01 (-0.01, 0.03)

[a]Fully adjusted for maternal parity (primiparous/multiparous), age at birth, ethnicity (Dominican/African American), receipt of public assistance (yes/no), completed high school (yes/no), partnership status (partnered/single), and linear and quadratic child age at visit (centered at age 5) and child sex with inverse probability weighting for loss to follow-up

[b]P-value Thresholds: ***P≤0.001; **P≤0.05, *P≤0.1

[c]65th Percentile: BDE-47: ≥42.1 ng/mL, BDE-99: ≥8.72 ng/mL, BDE-100: ≥7 ng/mL, BDE-153: ≥6.25 ng/mL, ΣPBDE: ≥64.2 ng/mL

[d]90th Percentile: BDE-47: ≥139 ng/mL, BDE-99: ≥35.1 ng/mL, BDE-100: ≥24.7 ng/mL, BDE-153: ≥14.4 ng/mL, ΣPBDE: ≥201 ng/mL

Supplemental Table S7. Sensitivity analyses for associations between cord plasma PBDEs and trajectories of child BMI z-score from 5 – 14 years.

	Beta Coefficient (95% Confidence Interval)[a,e]		
Predictor Variables	Model 3[b]	Model 4[c]	Model 5[d]
BDE-47			
BDE-47 ≥ 78 ng/mL	-0.22 (-0.55, 0.11)	-0.09 (-0.42, 0.24)	-0.11 (-0.51, 0.3)
Centered Age	0.12 (0.07, 0.16)***	0.14 (0.09, 0.18)***	0.07 (0.02, 0.12)**
Centered Age^2	-0.01 (-0.02, -0.01)***	-0.02 (-0.02, -0.01)***	-0.01 (-0.01, 0)*
BDE-47*Centered Age	0.02 (-0.09, 0.12)	-0.09 (-0.19, 0.01)*	0.004 (-0.13, 0.12)
BDE-47*Centered Age^2	0 (-0.02, 0.01)	0.01 (0, 0.03)	0.002 (-0.02, 0.02)
BDE-99			
BDE-99 ≥ 16.8 ng/mL	0.02 (-0.33, 0.36)	0.12 (-0.22, 0.45)	0.05 (-0.35, 0.44)
Centered Age	0.12 (0.08, 0.17) ***	0.13 (0.08, 0.18) ***	0.06 (0.01, 0.11)**
Centered Age^2	-0.01 (-0.02, -0.01) ***	-0.01 (-0.02, -0.01) ***	-0.01 (-0.01, 0)*
BDE-99*Centered Age	-0.04 (-0.15, 0.06)	-0.05 (-0.16, 0.05)	0.004 (-0.11, 0.12)
BDE-99*Centered Age^2	0 (-0.01, 0.02)	0.003 (-0.01, 0.02)	0 (-0.02, 0.02)
BDE-100			
BDE-100 ≥ 10.5 ng/mL	-0.05 (-0.38, 0.29)	-0.01 (-0.36, 0.34)	0.05 (-0.36, 0.45)
Centered Age	0.14 (0.09, 0.18)***	0.15 (0.1, 0.19)***	0.08 (0.03, 0.13)***
Centered Age^2	-0.02 (-0.02, -0.01)***	-0.02 (-0.02, -0.01)***	-0.01 (-0.02, 0)**
BDE-100*Centered Age	-0.1 (-0.2, 0)*	-0.13 (-0.24, -0.02)**	-0.07 (-0.19, 0.05)
BDE-100*Centered Age^2	0.02 (0, 0.03)**	0.02 (0, 0.04)**	0.01 (0, 0.03)
BDE-153			
BDE-153 ≥ 8.67 ng/mL	-0.16 (-0.52, 0.21)	-0.16 (-0.52, 0.2)	-0.09 (-0.55, 0.37)
Centered Age	0.13 (0.08, 0.17)***	0.13 (0.08, 0.18)***	0.09 (0.04, 0.13)***
Centered Age^2	-0.02 (-0.02, -0.01)***	-0.02 (-0.02, -0.01) ***	-0.01 (-0.02, 0)**
BDE-153*Centered Age	-0.05 (-0.15, 0.06)	-0.05 (-0.18, 0.07)	-0.12 (-0.25, 0.01)*
BDE-153*Centered Age^2	0.01 (0, 0.03)*	0.01 (-0.01, 0.03)	0.02 (0, 0.04)*
ΣPBDE			
ΣPBDE ≥ 112 ng/mL	-0.18 (-0.52, 0.16)	-0.03 (-0.35, 0.3)	-0.13 (-0.54, 0.28)
Centered Age	0.12 (0.07, 0.16)***	0.14 (0.09, 0.18)***	0.06 (0.01, 0.11)**
Centered Age^2	-0.01 (-0.02, -0.01)***	-0.02 (-0.02, -0.01)***	-0.01 (-0.01, 0)
ΣPBDE*Centered Age	0.01 (-0.1, 0.11)	-0.1 (-0.2, 0)*	0.02 (-0.1, 0.14)
ΣPBDE*Centered Age^2	0.002 (-0.02, 0.01)	0.01 (0, 0.03)*	0 (-0.02, 0.01)

[a]Beta coefficients are interpreted as the change in BMI Z-score when each PBDE is increased from low (< 80th percentile) to high (>80th percentile)
[b]Model 3: Fully adjusted for maternal parity (primiparous/multiparous), age at birth, ethnicity (Dominican/African American), receipt of public assistance (yes/no), completed high school (yes/no), partnership status (partnered/single), and linear and quadratic child age at visit (centered at age 5) and child sex with inverse probability weighting for loss to follow-up.
[c]Model 4: Fully adjusted models using lipid adjusted PBDEs
[d]Model 5: Additionally adjusted for PBDEs measured at year 7 (n=194)
[e]P-value Thresholds: ***P≤0.001; **P≤0.05, *P≤0.1
